# A systematic review of cases of meningitis in the absence of cerebrospinal fluid pleocytosis on lumbar puncture

**DOI:** 10.1186/s12879-019-4204-z

**Published:** 2019-08-05

**Authors:** Michelle Troendle, Alexis Pettigrew

**Affiliations:** 1 Division of Clinical Toxicology, Attending Physician, Virginia Commonwealth University, 1250 East Marshall St., P.O. Box 980401, Richmond, VA 23298-0401 USA; 2 AAPettigrew, LLC, 2345 Rock Branch Ln, North Garden, VA 22959 USA

**Keywords:** Meningitis without CSF pleocytosis, Cerebrospinal fluid pleocytosis, Cerebrospinal fluid culture

## Abstract

**Background:**

Definitive diagnosis of meningitis is made by analysis of cerebrospinal fluid (CSF) culture or polymerase chain reaction (PCR) obtained from a lumbar puncture (LP), which may take days. A timelier diagnostic clue of meningitis is pleocytosis on CSF analysis. However, meningitis may occur in the absence of pleocytosis on CSF.

Areas of Uncertainty: A diagnosis of meningitis seems less likely without pleocytosis on CSF, leading clinicians to prematurely exclude this. Further, there is little available literature on the subject.

**Methods:**

Ovid/Medline and Google Scholar search was conducted for cases of CSF culture-confirmed meningitis with lack of pleocytosis. Inclusion criterion was reported cases of CSF culture-positive or PCR positive meningitis in the absence of pleocytosis on LP. Exclusion criteria were pleocytosis on CSF, cases in which CSF cultures/PCR were not performed, and articles that did not include CSF laboratory values.

**Results:**

A total of 124 cases from 51 articles were included. Causative organisms were primarily bacterial (99 cases). Outcome was reported in 86 cases, 27 of which died and 59 survived. Mortality in viral, fungal and bacterial organisms was 0, 56 and 31%, respectively. The overall percentage of positive initial CSF PCR/culture for viral, fungal and bacterial organisms was 100, 89 and 82%, respectively. Blood cultures were performed in 79 of the 124 cases, 56 (71%) of which ultimately cultured the causative organism. In addition to bacteremia, concomitant sources of infection occurred in 17 cases.

**Conclusions:**

Meningitis in the absence of pleocytosis on CSF is rare. If this occurs, causative organism is likely bacterial. We recommend ordering blood cultures as an adjunct, and, if clinically relevant, concomitant sources of infection should be sought. If meningitis is suspected, empiric antibiotics/antifungals should be administered regardless of initial WBC count on lumbar puncture.

**Electronic supplementary material:**

The online version of this article (10.1186/s12879-019-4204-z) contains supplementary material, which is available to authorized users.

## Background

Meningitis is a serious acute infection of the meninges that can be caused by bacteria, viruses, parasites, or fungi [1]. Definitive diagnosis is made by analysis of cerebrospinal fluid (CSF) culture or polymerase chain reaction (PCR) in viruses obtained from a lumbar puncture (LP), which may take days. Timelier diagnostic clues of meningitis on CSF analysis include an elevated white blood cell (WBC) count or protein concentration, and decreased glucose concentration relative to blood [1–3]. However, there are reports of PCR/culture-proven meningitis in the absence of elevated WBC on CSF analysis (pleocytosis) [4–58].

The purpose of this paper is to review the published literature to describe themes associated with CSF culture-proven meningitis or CSF PCR positive in the absence of pleocytosis on initial LP. There are no conflicts of interest in this report.

Throughout this paper, several abbreviations are used, which include: AIDS - Acquired Immune Deficiency Syndrome; CSF - cerebrospinal fluid; LP - lumbar puncture; PCR - polymerase chain reaction; WBC - white blood cell; Χ^2^- chi-square.

## Methods

We conducted a search of Ovid/Medline and Google Scholar using a combination of keywords and controlled vocabulary representing the concepts “meningitis,” “cerebrospinal fluid,” and “absence of pleocytosis.” The reference lists of each included article were also searched for relevant citations not identified in the search.

All studies were reviewed by title and abstract and then by full text. Studies were included in this paper if they reported cases of CSF culture/PCR positive meningitis in the absence of pleocytosis on LP. Absence of pleocytosis was defined as CSF WBC ≤ 19 cells/mm^3^ in cases 28 days of age or younger, CSF WBC ≤ 9 cells/mm^3^ for cases 29–56 days of age, and CSF WBC ≤ 5 cells/mm^3^ in cases over 57 days of age [59]. Exclusion criteria were pleocytosis, defined as CSF WBC > 19 cells/mm^3^ in cases 28 days of age or younger, > 9 cells/mm^3^ for cases 29–56 days of age and CSF WBC > 5 cells/mm^3^ in cases over 57 days of age; other exclusion criteria were cases where CSF cultures were not performed, and articles that did not include CSF laboratory values.

**Fig. 1:**
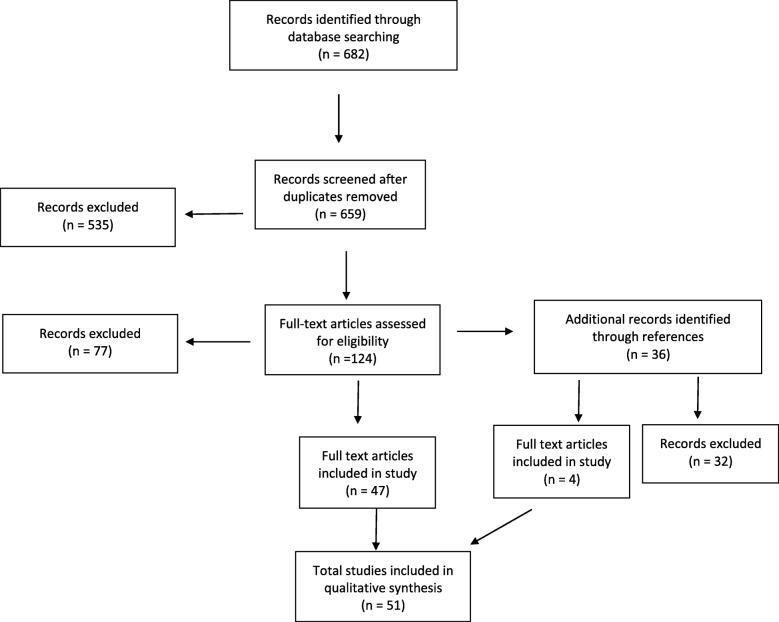
Flow diagram of articles excluded and included in study

We did not exclude based on age. Thus, this literature review encompasses patients of all ages, including neonates. The review includes both immunocompetent and immunosuppressed patient populations. We did not place restrictions on the years that articles were published. Articles in all languages were reviewed with the assistance of translators when indicated.The Ovid/Medline search yielded a total of 682 articles, 23 of which were duplicates. Of these articles, 535 were excluded as they were not found to be relevant by title or abstract. The remaining articles were assessed full-text for possible inclusion. Of these 124 remaining articles assessed by full text, 77 were excluded. Titles from references of the 124 articles screened by full text were also assessed for possible inclusion. We reviewed 36 articles from references, 4 of which met inclusion criteria. A total of 4 articles from references of full-text screened articles and 47 articles from Ovid/Medline search were included for a total of 51 articles (Fig. 1). Articles ranged in publication from 1972 to 2017.

**Table 1:** Demographics of age by gender, pathogen, co-morbidity and outcome

	Age Groups				
	Neonate (*n* = 17)	Pediatric (*n* = 61)	Adult (*n* = 36)	Senior (*n* = 10)	*p*-value
Gender					
Male (*n* = 43)	2 (12%)	17 (28%)	18 (50%)	6 (60%)	< 0.05
Female (*n* = 41)	1 (6%)	18 (30%)	18 (50%)	4 (40%)	
Unknown (*n* = 40)	14 (82%)	26 (43%)	0 (0%)	0 (0%)	
Pathogen					
Bacterial (*n* = 99)	17 (100%)	46 (75%)	26 (72%)	10 (100%)	< 0.05
Fungal (n = 9)	0 (0%)	1 (2%)	8 (22%)	0 (0%)	
Viral (*n* = 16)	0 (0%)	14 (23%)	2 (6%)	0 (0%)	
Co-morbidity					
Presence of Co-morbidities (*n* = 22)	0 (0%)	4 (7%)	16 (44%)	2 (20%)	< 0.05
Outcome					
Died (*n* = 27)	0 (0%)	10 (16%)	14 (39%)	3 (30%)	< 0.05
Survived (*n* = 59)	4 (24%)	33 (54%)	17 (47%)	5 (50%)	
Unknown (*n* = 38)	13 (76%)	18 (30%)	5 (14%)	2 (20%)	

There were 218 cases from the 51 articles which were reviewed for possible inclusion. Of these 218 cases, 124 met inclusion criteria. The 51 included articles include 24 case reports, 1 case report with literature review of bacterial meningitis in the absence of pleocytosis, 1 case report with retrospective chart review of cases of H. influenzae meningitis, 14 case series, 1 case series with literature review, 6 retrospective chart reviews, and 4 prospective cohort studies chart review. Characteristics of cases were summarized with descriptive statistics and 95% confidence intervals where appropriate. Mortality and percentage of positive initial CSF cultures/PCR were abstracted using a gold standard of all cases with final CSF culture/PCR proven meningitis.

As the above methods involved extrapolating cases from cases reported in the literature, the cases contained no personal data with no means to contact. As we conducted no experimentation on patients but made observations from already available data, informed consent, patient consent for publication, Institutional Review Board approval and Ethics Committee approval were not obtained.

## Results

Of the 218 cases initially identified, 124 had an absence of pleocytosis with culture proven meningitis. Additional file 1, located at the end of the article, summarizes our findings in term of age, gender, LP findings, pathogen, blood culture results, co-morbidities and outcome in terms of death, survival or unknown.

**Table 2:** Demographics of type of pathogen by gender, co-morbidities and outcome

	Type of Pathogen			
	Bacterial (*n* = 99)	Fungal (n = 9)	Viral (*n* = 16)	p-value
Gender				
Male (*n* = 43)	37 (37%)	5(56%)	1 (6%)	< 0.05
Female (*n* = 41)	33 (33%)	4 (44%)	4 (25%)	
Unknown (*n* = 40)	29 (29%)	0 (0%)	11 (69%)	
Comorbidities				
Presence of Co-morbidities (n = 22)	14 (14%)	8 (89%)	0 (0%)	< 0.05
Outcome				
Died (n = 27)	22 (22%)	5 (56%)	0 (0%)	< 0.05
Survived (n = 59)	50 (51%)	2 (44%)	5 (31%)	
Unknown (n = 38)	27 (27%)	0 (0%)	11 (69%)	

All included cases presented with a clinical suspicion of meningitis. While there was variation among cases, presenting symptoms included headache, fever, seizure, mental status changes, vomiting, neck stiffness and photophobia [4–57].

Tables 1 and 2 describe the demographics of the patients. Breakdown of age (by gender, pathogen, co-morbidity and outcome) and type of pathogen (by gender, presence of comorbidities and outcomes) are included. Chi-square analysis (Χ^2^) was performed to compare categorical data. Age ranged from the first day of life to 86 years. We defined neonate from age 1–28 days, pediatric 29 days – 17 years, adult 18–64 years, and senior as 65 years and over. Forty-one cases were female, 43 cases were male, and 40 cases did not have gender reported. Across all age groups, most cases were bacterial (99 of 124). However, viral and fungal infections also caused 16 and 9 cases, respectively. The most common bacterial organisms reported were *N. meningitides* (25 cases), *S. pneumoniae* (22 cases), and *H. influenzae* (17 cases). The most commonly reported viral organism was *Enterovirus* (12 cases), and the most commonly reported fungal organism was *Cryptococcus sp* (7 cases). Males were more likely to present with bacterial and fungal meningitis, while females were more likely to present with viral meningitis

All cases had no pleocytosis on the initial LP. A repeat LP was performed in 37 of the 124 cases, and WBC on LP was reported in 33. Four of these 33 cases (12%) still did not yield CSF pleocytosis. The organisms involved in this phenomenon were *N. meningitides, E. coli, Cryptococcus, and S. Aureus.*

Outcome in terms of death, survival, or unknown is summarized in Table 3. Outcome was reported in 86 of the 124 cases. Twenty-seven patients died, yielding a mortality of 33%.

**Table 3:** Outcome by Organism

*Bacterial*				
Organism	Died	Survived	Unknown	Mortality
* S. pneumoniae*	6	12	4	33%
* N. meningitidis*	7	14	4	33%
* H. influenzae*	3	11	3	21%
* L. monocytogenes*	1	1	1	50%
* Acenobacter*	0	0	1	N/A
* C. septicum*	1	0	0	100%
* Clostridium* sp.	1	0	0	100%
Bacteroides sp				
* E. coli*	2	4	1	33%
* Enterbacter*	0	0	1	N/A
* Enterococcus*	0	0	2	N/A
* Gram + Cocci*	0	0	3	N/A
Group B *Streptococcus*	0	3	3	0%
* M. polymorpha*	0	1	0	0%
* M. tuberculosis*	0	2	0	0%
* P. aeruginosa*	0	0	2	N/A
* P. Mirabilis*	1	0	0	100%
* S. aureus*	0	1	1	0%
* S. bovis*	0	1	0	0%
* S. parasanguinis on 1st culture, S. pneumoniae on 2nd culture*	0	0	1	N/A
*Fungal*				
Organism	Died	Survived	Unknown	Mortality
*Cryptococcus*	4	3	0	57%
*A. niger*	1	0	0	100%
*C. albicans*	0	1	0	0%
*Viral*				
Organism	Died	Survived	Unknown	Mortality
*Enterovirus*	0	1	11	0%
*Echovirus type 3*	0	1	0	0%
*Echovirus type 9*	0	1	0	0%
*Adenovirus type 3*	0	1	0	0%
*Herpes Simplex 1*	0	1	0	0%

**Table 4:** Percentage of Positive Results on 1st CSF Culture, and Results of Blood Cultures by Organism

Organism	*Bacterial*						
	1st CSF culture			Blood culture			
				Positive		Negative	Unknown
	Positive	Negative	Sensitivity	Total	Initial		
*S. pneumoniae*	18	4	82%	12	12	3	7
*N. meningitidis*	21	4	84%	16	16	6	3
*H. influenzae*	12	5	71%	12	9	1	4
*L. monocytogenes*	1	2	33%	3	3	0	0
*Acenobacter*	1	0	100%	0	0	0	1
*C. septicum*	1	0	100%	1	1	0	0
*Clostridium* sp.	1	0	100%	1	1	0	0
Bacteroides sp							
*E. coli*	5	2	71%	6	6	0	1
*Enterbacter*	1	0	100%	0	0	1	0
*Enterococcus*	2	0	100%	0	0	2	0
*Gram + Cocci*	3	0	100%	1	1	2	0
Group B *Streptococcus*	6	0	100%	2	2	2	2
*M. polymorpha*	1	0	100%	0	0	0	1
*M. tuberculosis*	1	1	50%	0	0	0	2
*P. aeruginosa*	2	0	100%	0	0	2	0
*P. Mirabilis*	1	0	100%	1	1	0	0
*S. aureus*	2	0	100%	0	0	2	0
*S. bovis*	1	0	100%	0	0	0	1
*S. parasanguinis on 1st culture, S. pneumoniae on 2nd culture*	1	0	N/A	0	0	0	1
Organism	*Fungal*						
				Blood culture			
	1st CSF culture			Positive			
	Positive	Negative	% Positive	Total	Initial	Negative	Unknown
*Cryptococcus*	6	1	86%	1	1	0	6
*A. niger*	1	0	100%	0	0	0	1
*C. albicans*	1	0	100%	0	0	1	0
Organism	*Viral*						
				Blood culture			
	1st CSF PCR			Positive			
	Positive	Negative	% Positive	Total	Initial	Negative	Unknown
*Enterovirus*	12	0	100%	0	0	0	12
*Echovirus type 3*	1	0	100%	0	0	0	1
*Echovirus type 9*	1	0	100%	0	0	0	1
*Adenovirus type 3*	1	0	100%	0	0	0	1
*Herpes Simplex 1*	1	0	100%	0	0	1	0

Mortality varied between types organisms. Across the age groups, adults were most likely to die. No patient that was afflicted with viral meningitis died. Fungal organisms yielded the highest mortality at 56%. Of the fungal infections, *Cryptococcus sp* was the most commonly reported organism (7 of 9 total fungal cases), with mortality in 57% of these cases. Further, these patients most likely had co-morbidities. The overall reported mortality of bacterial infections was 31%. The four most commonly reported bacterial organisms were *N. meningitides*, *S. pneumoniae*, *H. influenzae*, and *E. coli*, with mortality at 33, 33, and 21%, and 33%, respectively. There was one reported case dual infection by *Clostridium & Bacteroides* sp., which resulted in death.

Table 4 describes the sensitivity of CSF cultures/PCR on initial LP and the percentage of initial positive blood cultures. By the time of the second lumbar puncture, CSF culture/PCR returned positive for all cases. As CSF culture/PCR provides definitive diagnosis of meningitis, we assessed the sensitivity of positive initial CSF culture/PCR. The CSF culture/PCR on initial LP was positive for the causative organism in 105 of the 124 cases, yielding a percentage of positive initial CSF of 85% (95% CI of 77–90%). The overall percentage of positive initial CSF PCR for viral causes was 100%, and the positive initial culture for fungal and bacterial organisms was 89 and 82%, respectively.

There were 19 cases in which the initial CSF culture was negative. In 13 of these cases, blood cultures were taken at the time of the initial LP, and 11 were positive on initial blood draw. The 2 with initially negative blood cultures returned positive on repeat blood culture, and both cultured *H. influenzae*.

Blood cultures were performed in 79 of the 124 cases. Of these 79 cases, 56 (71%) ultimately cultured the causative organism. Fifty-five were bacterial, and one was fungal. Fifty-three cultured the causative organisms at the time of the initial blood culture. The 3 blood cultures that did not initially return positive were all the bacterium *H. influenzae*.

In addition to blood cultures, concomitant sources of infection for causative organism were reported in 17 cases (Table 5). There were 11 cases with a Chest x-ray (CXR) which was positive for pneumonia or pleural effusion, 8 of which cultured *S. pneumoniae*. There were 3 cases reported in which patients had arthrocentesis which cultured the same causative organism on CSF. One patient had a positive urine culture, which grew *P. mirabilis*. This patient had a co-morbidity of a chronic indwelling foley catheter which was clogged on initial presentation. Lastly, there were two cases of peritoneal fluid which cultured *E. coli*. Both had a history of alcohol abuse. In these 17 cases, if blood cultures were performed, they were positive except for the case of *C. albicans*.

**Table 5:** Cases with concomitant sources of infection

Age	Gender	CSF Culture	Blood culture	Outcome
Pneumonia or pleural effusion				
57 years	M	*S. pneumoniae*	*S. pneumoniae*	Survived
86 years	M	*S. pneumoniae*	*S. pneumoniae*	Death
18 years	M	*S. pneumoniae*	*n/a*	Death
62 years	M	*S. pneumoniae*	n/a	Death
59 years	M	*A. niger*	n/a	Death
50 years	F	*S. pneumoniae*	*S. pneumoniae*	Survived
2.5 months	M	*N. meningitidis*	n/a	Survived
1 year	F	*H. influenzae*	*H. influenzae*	Died
66 years	M	*S. pneumoniae*	*S. pneumoniae*	Survived
6 months	F	*S. pneumoniae*	n/a	Survived
50 years	F	*S. pneumoniae*	*S. pneumoniae*	Unknown
Arthrocentesis				
83 years	F	*N. meningitidis*	*N. meningitidis*	Survived
26 years	M	*H. influenzae*	*H. influenzae*	Survived
37 days	F	*C. albicans*	Negative	Survived
Urine				
76 years	F	*P. Mirabilis*	*P. mirabilis*	Death
Peritoneal fluid				
54 years	F	*E. coli*	*E. coli*	Death
63 years	M	*E. coli*	*E. coli*	Death

As above, the WBC on LP was within normal limits at the time of the first lumbar puncture. Other CSF markers that could indicate meningitis is elevated protein and depressed CSF glucose/serum glucose. In adults, the generally accepted normal range for protein in CSF is 15–45 mg/dL [59]. Unfortunately, there is no consensus in patients younger than 56 days of age as the blood-brain barrier has not fully formed. A study which specially looked at CSF protein markers in patients less than 56 days of yielded the 95th percentile values of protein in CSF as 115 mg/dL for infants ≤28 days and 89 mg/dL for infants 29–56 days [60]. Therefore, we used these cut-offs for this patient population, and any protein level above this was considered abnormal. Meningitis can also be further suspected if the CSF glucose/serum glucose is < 0.5 in age > 56 days and < 0.6 in age 1–56 days [59].

We analyzed the serum glucose and protein level in all patients to assess if there were instances if there was normal CSF markers for WBC, CSF glucose, and CSF protein. In our study, of the cases in which all markers were recorded, all CSF markers were within normal limits in 3 cases aged 1–56 days, 24 cases aged 56 days – 18 years, 12 adult patients, and 1 senior patient.

## Discussion

Meningitis in the absence of pleocytosis on CSF is rare. However, it has been reported in both adult and pediatric patients.

While most cases had CSF pleocytosis on repeat LP, 4 cases still had lack of pleocytosis on repeat lumbar puncture. In the case of *N. meningitides,* the patient had a co-morbidity of diabetes mellitus and positive blood culture and joint fluid culture. The patient with *E. coli* suffered from chronic ethanol abuse and had a positive blood and peritoneal fluid culture. We suspect that in these 2 cases meningitis developed secondary to seeding of the bacterium from another source, and this delay in time in addition to a chronic immune compromised state may have resulted in the patient being unable to mount an immune response at the time of repeat LP. This is further supported by both patients having a negative initial CSF culture, indicating that the organisms may have not truly inoculated the CSF at the time of initial LP. The patient with *Cryptococcus sp* suffered from acquired immune deficiency syndrome (AIDS), and likely the patient’s immunocompromised state prevented proper leukocytosis. Lastly, the patient with *S. aureus* had a history of congenital hydrocephalus with VP shunt with revision. It is unclear why this patient did not mount a leukocytosis on repeat LP.

Mortality varied between organisms, which may be due to several reasons. Fungal infection portended the poorest prognosis with an overall mortality in 56% of cases. All 7 cases with *Cryptococcal* meningitis were immunocompromised (5 with Acquired Immune Deficiency Syndrome (AIDS)), 1 with lupus with CD4 count of 75 yet but negative for Human Immunodeficiency Virus, and 1 with stage IV Hodgkin’s Lymphoma with pancytopenia), which likely increased mortality. Further, empiric coverage for meningitis may have targeted bacterial infections and not fungal infections, further increasing mortality in this subset. Viral infection portended the best prognosis, which is likely reflective of viral meningitis tending to be self-limited with good outcome [59]. While *Herpes simplex* virus is a notable exception, the one reported case survived [25, 59]. However, as this was reported in only one case, it is difficult to draw clinical conclusions. The overall reported mortality of bacterial infections was 31%. Lower mortality in bacterial infection than fungal infection may be secondary to empiric antibiotic administration and lower likelihood of immunocompromised state. While bacterial meningitis mortality was lower than fungal mortality, the mortality of 31% is nevertheless a poor outcome. Increased mortality may be attributed to delay in antibiotic administration secondary to normal appearing LP results. The one case of dual bacterial infection died. While this phenomenon occurred in only 1 patient, it is possible that the dual infection may have increased mortality.

Ultimately, 100% of CSF cultures returned as positive at the time of second lumbar puncture. As above, the initial CSF cultured positive for causative organism in 105 of the 124 cases (85%). There are several possibilities why the initial CSF culture would have been negative. First, patients may have received IV antibiotics prior to LP, temporarily sterilizing the CSF and resulting in a negative culture. Second, the CSF draw might not have been sufficient to culture. Third, patients may have developed meningitis secondary to another source such as bacteremia, which had not seeded into the CSF prior to the time of the initial LP [61]. Fourth, immunocompromised state may have hindered pleocytosis. Lastly, the patient may have had a fulminant course of meningitis, which did not give the patient time to mount a proper immune response.

Blood cultures had greatest clinical utility with bacterial infection. Only 1 bacterium, *H. influenzae*, had no growth on the initial blood culture. We hypothesize that the bacterium had not yet seeded into the bloodstream from the original source of infection at the time of initial blood culture. If clinically relevant, other sources of infection, such as joint fluid, peritoneal fluid, pleural fluid and urine should be cultured for possible causative organism.

### Limitations

We recognize several limitations in this literature review. While to our knowledge this is the largest review of available literature of meningitis in the absence of pleocytosis, is likely that this phenomenon is underreported. One possible cause of underreporting is that these cases meningitis may be overlooked due to seemingly normal appearing CSF biochemistry. Thus, our sample size is still relatively small, and results may not be generalizable. Further, some articles did not present all data we sought to analyze. We also recognize that viral meningitis without pleocytosis may be classified as headache and no further CSF analysis performed. If confirmatory CSF PCR is not performed, underreporting will occur. As this was a literature review derived from pooled data, articles were subject to varying reported clinical information, methods, results, and timelines.

Another limitation of the study is that articles were subject to selection bias. One prospective study targeted *N. meningitides*, another *Enterovirus*, and another *Cryptococcus sp* in patients with AIDS, thus increasing the number of these organisms reported [20, 33, 44]. Two prospective cohort studies specifically analyzed meningitis in the absence of pleocytosis by patient age [27, 30]. One specifically analyzed neonates, and the other patients > 16 years of age [27, 30]. Thus, this not only skewed the age of the patients, but also the pathogens reported, as these vary with age. Lastly, vaccination of *H. influenzae B* in childhood has caused a decline in this organism. Many of the articles that reported this were written before vaccination, and, therefore, results may not reflect what is seen in current practice.

There were also a variety in the immunocompetent state of the patient. This study includes oncology patients who may have been neutropenic secondary to chemotherapy, AIDS patients who were immunocompromised secondary to CD4 deficiency, and patients who were immunocompromised due to extremes of age. Thus, laboratory findings, clinical course, morbidity and mortality in these populations may not be generalizable in an immunocompetent population.

Mortality and co-morbidities that may have affected it were also underreported. Lastly, the administration of antibiotics before LP, which could have caused negative cultures, could have occurred but was not reported.

## Conclusion

Although rarely reported, meningitis in the absence of pleocytosis may occur. While bacterial organisms are most commonly implicated, it may also occur in viral or fungal infections. It occurs in either gender nearly equally and may occur at any age range. Viral infection portends a favorable prognosis. Mortality may be increased with dual infection, fungal infection and immunocompromised state. Given the high mortality associated with bacterial and fungal meningitis in the absence of pleocytosis, clinicians should maintain a suspicion of meningitis in the absence of initial CSF pleocytosis when the clinical picture is highly suggestive and consider appropriate antibiotic/antifungal therapy while awaiting culture results and/or a repeat LP. It has been hypothesized that lack of pleocytosis may occur due to increased severity of disease with rapid progression, leaving insufficient time for an adequate inflammatory response to be mounted prior to obtaining CSF [62, 19]. Further, examination of other markers of the CSF, such as higher than expected CSF protein, high opening pressure, and depressed CSF glucose/serum glucose ratio may decrease likelihood of missed diagnosis of meningitis.

Since bacterial meningitis can occur secondary to underlying bacteremia from other sources, we suggest ordering blood cultures routinely in suspected cases. Blood cultures may need to be repeated, especially in cases of *H. influenzae*. CSF from initial LP may not culture the organism, and a repeat LP may be indicated to determine the causative organism. If meningitis is present, pleocytosis is likely to be present on repeat LP. However, if patients are severely immunocompromised, have significant co-morbidities, or have meningitis secondary to inoculation of organism from another primary source, a pleocytosis still may not occur. If clinical suspicion suggests meningitis in a patient who is exhibiting a rapid, fulminant course, we recommend empiric treatment for meningitis prior to performing the LP, as this course of action may be life-saving.

## Additional file

Additional file 1: Summary table of patient age, gender, CSF results, blood cultures and outcome [58]. (XLSX 35 kb)

## Data Availability

Availability of data and materials is included in reference section.

## Abbreviations

AIDS: Acquired Immune Deficiency Syndrome
CSF: Cerebrospinal fluid
LP: Lumbar puncture
PCR: Polymerase chain reaction
WBC: White blood cell
Χ^2^: Chi-square
